# The underlying mechanisms for severe COVID-19 progression in people with diabetes mellitus: a critical review

**DOI:** 10.3934/publichealth.2021057

**Published:** 2021-10-26

**Authors:** María D Figueroa-Pizano, Alma C Campa-Mada, Elizabeth Carvajal-Millan, Karla G Martinez-Robinson, Agustin Rascon Chu

**Affiliations:** Research Center for Food and Development, CIAD, AC, Carretera Gustavo Enrique Astiazarán Rosas No. 46, C.P. 83304, Hermosillo, Sonora, México

**Keywords:** COVID-19, diabetes mellitus, chronic inflammation, impaired immune system, SARS-CoV-2 receptor, ACE2 polymorphisms, diabetogenic effect

## Abstract

Diabetes mellitus (DM) has a high incidence of comorbidities among patients with severe coronavirus disease 2019 (COVID-19). The elevated prevalence of DM in the world population makes it a significant risk factor because diabetic individuals appear to be prone to clinical complications and have increased mortality rates. Here, we review the possible underlying mechanisms involved in DM that led to worse outcomes in COVID-19. The impacts of hyperglycemia side effects, secondary comorbidities, weakened innate and adaptive immunity, chronic inflammation, and poor nutritional status, commonly present in DM, are discussed. The role of the SARS-CoV-2 receptor and its polymorphic variations on higher binding affinity to facilitate viral uptake in people with DM were also considered. Clinical differences between individuals with type 1 DM and type 2 DM affected by COVID-19 and the potential diabetogenic effect of SARS-CoV-2 infection were addressed.

## 1. Introduction

For an entire year, the world's population has been impacted by the pandemic caused by coronavirus disease 2019 (COVID-19). Many articles describe COVID-19 as a viral infection produced by severe acute respiratory syndrome coronavirus 2 (SARS-CoV-2), a new coronavirus strain [1]. The first cases of COVID-19 were detected in Wuhan, Hubei Province, China, in December 2019. Then, several countries were affected, forcing the World Health Organization (WHO) to declare, in March 2020, COVID-19 as a pandemic. According to the “COVID-19 Dashboard by the Center for Systems Science and Engineering (CSSE) at Johns Hopkins University”, in March 2021, 192 countries around the world presented active cases of this disease, with a total of confirmed cumulative instances of 131,696,594 and 2,859,357 deaths. The United States, Brazil, and India have been the most affected countries, with more than 30, 13, and 12 million confirmed cases, respectively. Mexico could be considered one of the countries most damaged by COVID-19 due to the high case-fatality ratio. At the time of writing this review, Mexico presented a total of 204,147 fatal cases, behind only the United States (555,403) and Brazil (332,752), even though it has only presented approximately 2 million confirmed cases. It is essential to mention that DM has a high prevalence in Mexico (10% in 2016) and is one of the leading causes of death [2],[3]. Most likely, DM is a significant pre-existing condition shaping the impact of COVID-19 in this country triggered by syndemic interactions [4].

Many of the COVID-19 outbreaks have been slightly controlled due to lockdown and social distancing measures, and the application of new vaccines can dramatically drop COVID-19 cases around the world. However, new variants of SARS-CoV-2 with a faster spreading rate or increased virulence continue to threaten public health, especially for vulnerable populations [5]–[8]. Four strains have been classified by the WHO as variants of concerns (VOCs): the B.1.1.7 strain detected in the United Kingdom (labeled Alpha), the B.1.351 strain identified in South Africa (Beta), the P.1 strain in Brazil (Gamma), and the B.1.617 strain documented earliest in India (Delta). Other variants have been detected in the United States: the B.1.429 and B.1.526 strains from California and New York, respectively. A unique SARS-CoV-2 spike protein P681H variant was detected in Israel, but it was not associated with a high prevalence [9]. Although some of these variants can be mitigated (at least the severe outcomes) with a complete vaccination scheme, there only seems to have an effect for a limited time, which is likely occurring in the Israeli population, which is facing a fourth COVID-19 outbreak, although over 80% of its adult population received two doses of Pfizer BNT162b2 [10]. Several studies have proven the efficacy of the BNT162b2 vaccine against SARS-CoV-2 variants: B.1.1.7 and B.1.35 [11],[12]. The B.1.1.7 variant was predominant in the Israeli population (94.5%), but an observational study showed how vaccination helps to control the pandemic [13]. However, many cases have recently been active in fully vaccinated individuals, potentially indicating reduced vaccine effectiveness against VOCs within particular time frames [14].

Even though COVID-19 was initially not considered as dangerous as the previous diseases caused by a coronavirus (severe acute respiratory syndrome (SARS) and Middle East respiratory syndrome (MERS)) because its case fatality rate (CFR) was 4.2% (9.6% for SARS was and 34.4% for MERS), it has been highly contagious and easily transmissible between people [15]. This viral infection commonly spreads through droplet inhalation from sneezing, coughing, or direct contact with an infected individual [16]. Many people are asymptomatic (approximately 80%) and are only carriers, and others develop mild symptoms, such as fatigue, loss of smell and taste, dizziness, and headache [17]. Dry cough, fever, dyspnea, and diarrhea have also been reported among COVID-19 patients [18]. However, severe cases of COVID-19 frequently present severe respiratory problems, such as hypoxia and atypical pneumonia, and some cases can progress and be fatal because SARS-CoV-2 can affect vital organs and produce multiorgan failure [17],[19],[20]. The findings indicated that an exacerbated inflammatory status, called a “cytokine storm” is one of the leading causes that complicates long COVID-19 development [1]. Among the severe cases of COVID-19, old age and comorbidities, such as hypertension, obesity, cardiovascular diseases, lung diseases, kidney diseases, and diabetes, have been identified as the principal risk factors for the progression of disease severity [1],[21]–[23].

Diabetes mellitus (DM) is one of the major metabolic diseases globally, causing high morbidity and mortality indices, and it is considered a pandemic [24]. DM patients are also facing the current COVID-19 pandemic. The average prevalence of DM among patients with COVID-19 has been approximately 10–20% [25]–[28], similar to the general population (10–15%). However, it has varied by region, age, and ethnicity. According to statistical analyses, Asian countries have a lower prevalence of DM among COVID-19 patients than other countries [29], with rates ranging from 7.9% to 10% [21],[30] and, in a few cases, 15–17% [31]. In most European countries, DM in patients with COVID-19 is approximately 15–19% [32]–[34], and some of them, such as France, present DM in 23.6% [35]. In contrast, a whole-population study from England found only 5.2% DM between COVID-19 patients [36]. In American countries, the US presented the highest prevalence rates, approximately 12% to 30% [37],[38], while in Latin America, DM has been reported in 8.3% of COVID-19 cases [39]. Additionally, studies from London and the US said that nonwhite patients with DM were the majority among COVID-19 cohorts, with 72% and 56%, respectively [40],[41]. Furthermore, the prevalence of DM in COVID-19 patients has been higher in adults over 50 years of age, especially in those aged 65-70 years [29],[30],[33],[37],[42]. Other studies indicate that male patients with DM had a significant prevalence (50–60%) among COVID-19 patients [31],[42],[43]. Unfortunately, the association of DM with COVID-19 has usually resulted in severe illness or poor prognosis [21],[34],[44]. Frequently, these patients were more susceptible to severe COVID-19; they were admitted to the intensive care unit (ICU), assisted with mechanical ventilation, and suffered high in-hospital mortality (7–20%) compared with those cases of COVID-19 without DM (2%) [25],[43]–[48]. People with or without DM have presented hyperglycemia episodes during SARS-CoV-2 infection, and new-onset DM has been diagnosed in some post-COVID-19 cases [49]–[54]. Therefore, the question has been raised as to whether COVID-19 can cause DM.

**Figure 1.**
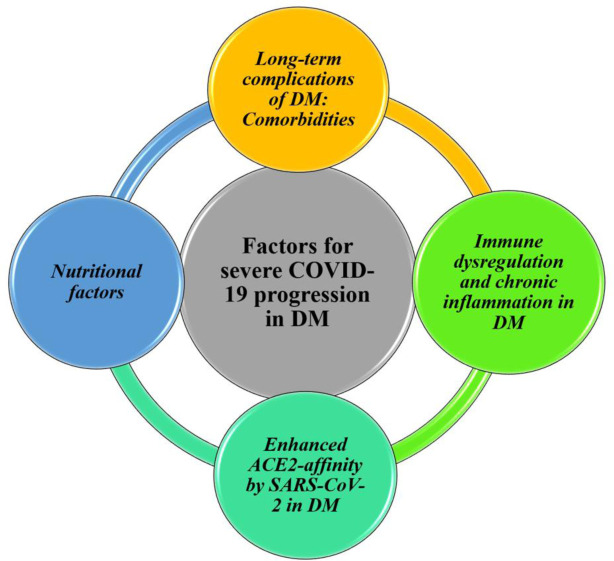
Main proposed underlying mechanisms for severe COVID-19 progression in people with DM.

The evidence on severe COVID-19 outcomes in people with DM includes statistical data, clinical characteristics, treatment and care, and the possible underlying mechanisms. The deteriorated health condition has been explained by the increased susceptibility of people with DM to severe COVID-19, involving hyperglycemia side effects, secondary comorbidities, weakened innate and adaptive immunity, chronic inflammation, and poor nutritional status [22],[55]–[58]. Additionally, SARS-CoV-2 may have a high binding affinity for ACE2 in DM, enabling accelerated viral uptake, which is complemented by the reduced clearance capacity [59],[60]. This review addresses and complements some of these points with the most recent findings in DM and COVID-19, expanding on the underlying mechanics (Figure 1). Particular interest is focused on the limited information about differences found between type 1 and type 2 DM and the possible diabetogenic effect of SARS-CoV-2.

## 2. Susceptibility of people with DM to develop severe COVID-19

It is not unexpected that infection by SARS-CoV-2 can cause critical problems in patients with DM because, in the last two coronavirus-related illnesses (SARS and MERS), these individuals were more vulnerable and presented more medical conditions [61]–[64]. In addition, DM carries different complications that weaken individuals' health, making it difficult for survivors to fight infections when pathogens invade them. Comorbidities associated with DM, low-grade inflammatory conditions, deteriorated immune response, and deficient nutritional status are the main factors that expose diabetic individuals to becoming seriously ill during infections.

### 2.1. Long-term complications associated with DM: comorbidities

DM is a chronic and metabolic disorder that affects the health of millions of individuals and can cause secondary complications over time. The main characteristic of DM is a constantly high blood glucose level (fasting blood glucose above 120 mg/dL or HbAc1 higher than 6.5%) due to the destruction of insulin-producing beta cells or insulin resistance [65],[66]. However, the pathophysiology of DM involves multiple factors, making it a complex disease. Generally, elevated glucose levels create abnormalities in lipid and protein metabolism, glucotoxicity, endothelial damage, and several microvascular and macrovascular complications, among others [67]–[70]. These pathologies damage vital organs and lead to typical complications of DM, such as cardiovascular disease, nervous system deterioration, and kidney disease [71]. Glucose dysregulation exposes patients to other health problems and makes them vulnerable to different conditions. However, there is no evidence that DM increases susceptibility to COVID-19; it is only considered a risk factor for severe progression of this infectious disease. Pre-existing diabetes in COVID-19 patients was associated with an almost double risk of developing acute COVID-19 symptoms and a three times increased risk of in-hospital mortality [29]. In particular, diabetic individuals with elevated glycosylated hemoglobin (HbAc1) levels, as a representation of poor glycemic control before infection, might have an increased COVID-19-related mortality rate [72].

COVID-19 patients with DM had higher comorbidity rates (hypertension, dyslipidemia, cardiovascular disease, and dementia) and presented more extended hospitalization and higher mortality than those without DM [46],[73],[74]. Sun et al. [75] found an increased mortality risk in COVID-19 patients with DM combined with hypertension. Sutter et al. [35] concluded that DM alone was not significantly associated with severe consequences in patients with COVID-19 in a French cohort. They compared the clinical results of individuals with and without DM hospitalized for COVID-19 using a propensity score-matching (PSM) method to avoid the effects of the common comorbidities presented in both diseases. The participants in both groups mainly showed hypertension (76.3%), a history of cardiovascular disease (32.8%), chronic kidney disease (22.1%), and chronic obstructive pulmonary disease (6.4%). Their results suggested that severe outcomes (admission to the ICU/in-hospital death) in diabetic patients with COVID-19 was mainly driven by comorbidities accompanying DM rather than DM itself. Notably, independent factors associated with the severe progression of COVID-19, such as hypertension, advanced age, and being male, have been frequently observed among diabetic patients who presented worse results due to COVID-19.

### 2.2. Impaired immune system and chronic inflammation in DM

**Figure 2.**
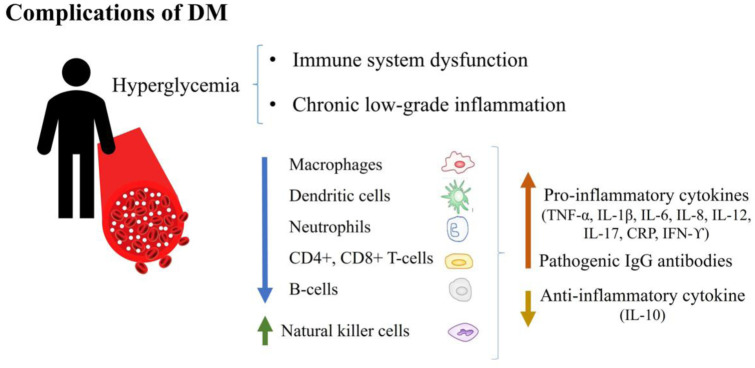
Immunological dysregulations in patients with DM.

An inflammatory condition and an impaired immune system are common complications of DM, as both caused by hyperglycemia (Figure 2) [76]. Long- or short-term hyperglycemia increases the formation of advanced glycation end products (AGEs), stimulating the production of reactive oxygen species (ROS), triggering inflammatory pathways [67]. Chronic low-grade inflammation in DM frequently causes immune system dysfunction, damping innate and adaptive immune responses [24],[77]. The cellular immune response of diabetic patients usually presents defects and dysfunction, including reduced activity and number of macrophages and dendritic cells. Neutrophils are activated but perform impaired phagocytosis and are susceptible to apoptosis [78]. Natural killer cells are increased and produce high cytokine levels, but they are prone to apoptosis [67],[77]. Adaptive immune cells, T lymphocytes (CD4+, CD8+), and B lymphocytes present reduced action and decreased proliferation caused by hyperglycemia [77],[79]. Specifically, both circulating and tissue-resident B lymphocytes from people with diabetes have displayed reduced secretion of the anti-inflammatory cytokine interleukin-10 and a reduced capacity to produce de novo antibody responses, even when they can produce a higher basal secretion of proinflammatory cytokines. Additionally, B lymphocytes in diabetic individuals have pathogenic IgG antibodies, causing an unfavorable effect [80]. Humoral components secreted by T and B lymphocytes, mainly proinflammatory mediators, such as tumor necrosis factor-α (TNF-α), interleukin (IL)-1β, IL-6, IL-8, IL-12, IL-17, C-reactive protein (CRP) and interferon-ϒ (IFN-ϒ), have presented elevated baseline levels in diabetic individuals [78]. Patients with type 2 DM exhibited a high prevalence (52%) of residual inflammatory risk, characterized by persistent circulating levels of high sensitivity CRP (>2 mg/L) despite optimal control of LDL cholesterol [81]. High glucose levels may also contribute to Langerhans destruction in islets, increasing the production and release of IL-1β. Then, NF-κB is activated, leading to Fas gene upregulation and provoking Fas-triggered apoptosis. IL-1β was observed in pancreatic sections of type 2 DM patients, implying that β-cells in the islets were the source of glucose-induced IL-1β production [82],[83]. These immunological dysregulations in patients with DM suggest a poor ability to control pathogens during infections.

The chronic inflammatory condition in DM is considered the underlying mechanism for the “cytosine storm” complication and worsens the outcomes of COVID-19 [84]. It is assumed that hyperinflammation may result in multiorgan failure, a general complication in critical COVID-19 cases. The hyperinflammatory event in patients with DM and COVID-19 has also been related to the downregulation of microRNA-146a. MicroRNA-146a is a natural regulator of the excessive inflammatory response to the virus; however, it is downregulated in individuals with DM, obesity, and hypertension [85]. Thus, the inflammatory reaction to SARS-CoV-2 “added” to chronic inflammation in DM and the lack of a feedback mechanism to limit the inflammatory response due to the downregulation of microRNA-146a in DM may lead to severe COVID-19 outcomes.

The risk factors for a fast progression of COVID-19 in individuals with DM involve clinical parameters with a more severe inflammatory status and lymphopenia. Proinflammatory molecules, such as IL-2, IL-4, IL-5, IL-6, IL-10, IL-13, TNF-α, IFN-ϒ, and CRP, were found at higher levels in COVID-19 patients with DM than in COVID-19 patients without DM [84],[86]–[88]. Cellular abnormalities were also present in these individuals, including elevated neutrophil count and a low quantity of lymphocytes [28],[89]–[91]. CD8+ cells, CD3+ cells, CD16+ cells, CD56 cells, CD19+ cells, and CD4+ cells were below the expected levels, suggesting an inadequate or compromised immune response in these individuals [44],[84],[89]. Correlation analyses are mainly associated with increased levels of IL-6 and CRP and decreased CD4+ cell counts with the severity of COVID-19 in people with diabetes [84],[92]. IL-6 is a pleiotropic cytokine with both pro- and anti-inflammatory properties. The proinflammatory activity of IL-6 is activated through soluble receptors (*trans-signaling*), causing monocyte recruitment and increasing oxidative stress [93]. The latter can damage proteins, lipids, DNA, and the cell's structure and function, likely leading to accelerated COVID-19 development. In addition, IL-6 regulates CRP production under transcriptional control, an acute phase response protein used to evaluate disease activity in inflammatory conditions [94].

High levels of D-dimer are another characteristic laboratory index in patients with diabetes affected by severe COVID-19 and are considered a predictor of poor prognosis [28],[86],[91],[95]. D-dimer results from fibrin degradation, and its values are a criterion for evaluating intravascular coagulation; an elevated concentration (>1 mg/L) of D-dimer is related to thrombosis activation [95]–[97]. Augmented platelet aggregation and activation can be present in DM, promoting a hypercoagulable prothrombotic state [98],[99]. This abnormality has also commonly been detected in fatal cases of COVID-19, adding to the previous complications of diabetic patients [18].

Diabetic patients with COVID-19 presented more ground-glass opacities in both lungs and showed more delayed virus clearance than those without DM. The lesions found in both lungs, obtained by chest computed tomography (CT), confirm acute inflammation in this organ caused by the cytokine storm [84],[88],[100]. Complementarily, individuals with severe COVID-19 and DM have shown increased lactate dehydrogenase levels (LHDs), which are used to indicate lung injury, implying severe lung damage [28],[46],[84],[101]. Dysregulation of other metabolites in COVID-19 patients with DM suggests that not only lungs are affected by SARS-CoV-2 infection. COVID-19 patients with DM present high urea and creatinine levels and low albumin levels, indicating acute kidney injury [28],[100]. Kidney malfunction is a common side effect in long-term diabetic patients, although infection with SARS-CoV-2 can exacerbate this problem. Guo 2021 et al. [101] reported that among diabetic patients with COVID-19, those with proteinuria were mainly admitted to the ICU, indicating significant nephropathy associated with COVID-19. Additionally, patients with DM admitted to the ICU had a longer recuperation time than those who were not admitted to the ICU and showed a slower lung lesion recovery [90],[91],[101],[102]. The slower SARS-CoV-2 clearance in diabetic patients was due to the impaired immune system or better viral entry produced by increased ACE2 [101],[103].

### 2.3. Enhanced affinity and entry of SARS-CoV-2 in DM patients

ACE2 is a transmembrane protein widely recognized for its function in the renin-angiotensin-aldosterone system (RAAS) and currently for its role as host receptor for SARS-CoV-2. In the RAAS, ACE2 acts as a carboxypeptidase hydrolyzing the vasoconstrictor angiotensin II into the vasodilator angiotensin-(1-7), aiding in arterial pressure regulation [104]–[106]. Eventually, ACE2 hydrolyzes, although with less efficacy, angiotensin I into angiotensin-(1-9), which is subsequently transformed into angiotensin-(1-7) by ACE1 [107]. The regulatory functions of ACE2 are not limited to the RAAS. In the gut, ACE2 is essential for expressing neutral amino acid transporters, maintaining intestinal amino acid homeostasis, controlling the expression of antimicrobial peptides, and regulating the ecology of the microbiome [108],[109]. Most likely, ACE2 has extensive biological activities that depend on tissue localization. ACE2 is naturally expressed in several vital organs, such as the lungs, intestines, kidneys, heart, pancreas, vascular endothelial cells, cerebral neurons, oral mucosa cells, and immune cells [110]–[114]. In addition, there is a soluble form of ACE2 without a membrane anchor found in low concentrations in blood [115]. In 2003, ACE2 was identified as the gateway for SARS-CoV into the human body; in 2019, it was established as the cellular host receptor for SAR-CoV-2 by binding the viral surface spike glycoprotein [116],[117]. However, the spike glycoprotein of SARS-CoV-2 presents a receptor-binding domain (RBD) with a superior binding affinity (10- to 20-fold) for ACE2 than the RBD of SARS-CoV [16],[118]. ACE2 binding with spike leads to fusion of the virion membrane with the host cell, allowing cell access to SARS-CoV-2 and RNA release in the cytoplasm [16],[118].

After ACE2 binding by SARS-CoV-2, ACE2 is inactivated and downregulated, causing a disturbance in RAAS regulation. A reduction in ACE2 activity implies angiotensin II accumulation and reduced angiotensin concentration (1-7). High levels of angiotensin II are associated with the triggering of hyperglycemia, a clinical feature frequently observed in patients with COVID-19 [119]. Although ACE2 is expressed ubiquitously in human tissues, not all ACE2-expressing organs are equally involved in COVID-19 pathophysiology, with the lungs and intestines being the principal targets [114],[120]. In addition, ACE2 expression in human tissues is modified by genetic factors, age, sex, obesity, and chronic diseases, affecting the SARS-CoV-2 infection rate and the chances of suffering from severe COVID-19 [120].

Regarding ACE2 modulation by chronic diseases, there is a controversial link between modification of ACE2 expression and DM development. While some studies note that DM can reduce kidney ACE2 expression, others indicate an increase in ACE2 in the diabetic kidney. According to Chen et al. [121], human data revealed reduced ACE2 expression in those with DM who received inflammatory cytokine treatment. Mizuiri et al. [122] found that diabetic kidney tissues, analyzed by immunohistochemistry and hybridization, presented decreased ACE2 expression and increased ACE1 expression compared to healthy individuals. The authors suggest that the high ACE1/ACE2 ratio in the kidneys of DM patients might contribute to renal injury. Reich et al. [123] found reduced expression of ACE2 mRNA (approximately 50%) and protein in renal biopsies of people with kidney disease caused by type 2 DM. Similar results were observed by Tikellis et al. [111] in the kidneys from diabetic Sprague–Dawley rats as both ACE2 mRNA and protein levels were decreased. In contrast, Gilbert et al. [124] found that ACE2 mRNA increased approximately 2-fold in diabetic patients' kidney biopsies compared to healthy control subjects. Soldo et al. [125] reported that ACE2 mRNA is upregulated in the livers of males, elderly individuals, and diabetic patients, situations related to unfavorable outcomes of COVID-19. In the lungs, ACE2 protein was more elevated in patients with type 2 DM than in healthy individuals [126]. As part of the RAAS, ACE2 produces the vasodilator angiotensin-(1-7), contributing to the control of hypertension. The ACE2 reduction in diabetic patients would likely complicate the disease and lead to critical outcomes of COVID-19. In contrast, the elevation of ACE2 could be associated with increased SARS-CoV-2 infection and angiotensin II accumulation, leading to acute damage in that ACE2-expressing organ to worsening COVID-19 outcomes.

Alternatively, it is commonly accepted that ACE inhibitors (ACEis) and angiotensin-receptor blockers (ARBs), drugs widely used for DM and hypertension, stimulate ACE2 expression. Thus, the frequent consumption of ACEis and ARBs would promote SARS-CoV-2 entry and increase the chances for severe COVID-19 [127],[128]. However, the clinical outcomes of COVID-19 were comparable between diabetic patients who used or did not use ACEis or ARBs [28]. Menon et al. [129] demonstrated that ACE2 expression levels in kidneys affected by DM were unaltered by exposure to RAAS inhibitors. A recent study by Batchu et al. [130] demonstrated ACE2 upregulation in the kidneys and lungs of diabetic mice, which is driven by comorbidities and not by RAAS blockade. They also proposed that upregulation of ACE2 activity in the lung may be conducive to severe COVID-19. In parallel, Gilbert et al. [124] noted no differences between transcripts (mRNA) of ACE2 in diabetic kidneys of recipients and nonrecipients of RAAS blockade agents. Simultaneously, Sriram et al. [131] observed that data collection from several human studies does not support the hypothesis that ACEi or ARB usage increases ACE2 expression and the risk for complications from COVID-19. They have even proposed continuing to use ACEis and ARBs in patients with DM and COVID-19.

ACE2 gene (*ACE2*) polymorphisms are essential in developing severe COVID-19 outcomes in DM patients. Human *ACE2* is situated on chromosome Xp22, contains 18 exons that encode the 805-amino acid ACE2 protein, and commonly presents variations in sequence [132]. Approximately 140 *ACE2* single nucleotide polymorphisms (SNPs) have been detected in the world population, hypothetically due to genetic adaptation to distinct climatic conditions. However, several SNPs of *ACE2* have also been identified as driving factors for hypertension, DM, cerebral stroke, or coronary artery disease because they modulate the RAAS pathway. In a Chinese study performed with type 2 DM individuals, the SNPs rs2074192 and rs714205 in *ACE2* were associated with susceptibility to diabetic retinopathy only in women [133]. Although none of the ACE2 polymorphisms studied in cohorts of type 1 DM from Finland and Ireland have been associated with diabetic nephropathy, it is not ruled out that they have a weak effect [134],[135]. The genetic polymorphism of G8790A in *ACE2* plays an essential role in the pathogenesis of type 2 DM, complemented with coronary heart disease (CHD), a common chronic complication in DM [136]. Similarly, in Caucasians with type 2 DM, rs2074192, rs4240157, rs4646188, and rs1978124 genetic variations in *ACE2* were associated with hypertension and reduced systolic function in men. Additionally, rs4240157 and rs1978124 were related to hypertension and an increased left ventricular mass in women [137].

Most likely, *ACE2* polymorphisms also determine SARS-CoV-2 entry into host cells by altering the amino acid sequence on ACE2, leading to either acceleration or inhibition of spike-protein binding [138]. *In silico* molecular docking has predicted six missense variants of *ACE2* with higher affinity for SARS-CoV-2 spike protein RBD than wild type *ACE* and 11 other variants with lower affinity [139]. An additional simulation study confirmed an increased association of the K26R polymorphism with the SARS-CoV-2 spike protein and a decreased attraction of the S19P *ACE2* variation [140]. However, another study excluded the S19P variant because it falls into the cleavage site of the ACE2 precursor, which could alter the mature protein [139]. In French-Canadian individuals, the SNP rs2074192 represents a risk factor for hypertension in adult obese adult men, and the most severe outcomes of COVID-19 were associated with this *ACE2* variation [141]. To date, ACE2 SNPs have not been related as a risk factor for DM and severe outcomes of COVID-19; however, hypertension is a relatively common secondary condition in DM. The effect of ACE2 variants on SARS-CoV-2 entrance may influence individuals' susceptibility to COVID-19 and impact final clinical outcomes [142]. ACE2 SNPs might cause different responses to ACE2 blockers used in COVID-19 patients because they strongly influence the function and stability of the ACE2 protein [143].

### 2.4. Nutritional factors

The degraded nutritional status that patients with diabetes may present due to a shortage of essential nutrients has been deemed an added condition for the poor prognosis of COVID-19. Vitamin B12 deficiency was proposed as an accomplice for severe outcomes of COVID-19 in the elderly and diabetic patients because its insufficiency may affect carbon metabolism for DNA syntheses [144]. In another study, 26.3% of critically ill diabetic patients infected by SARS-CoV-2 presented thiamine deficiency (<28 µg/L), compromising glucose metabolism and energy homeostasis [145]. Many studies have linked morbidity and mortality of COVID-19 patients with an indirect effect of vitamin D deficiency that impacts cytokine activity, ACE2 levels, and thrombosis [146]–[148]. Additionally, various observational studies have found vitamin D deficiency in DM [149],[150]. Severe COVID-19 complications in patients with DM are more significant when vitamin D levels are below 10 ng/mL; however, more convincing data on deficiency effects in the diabetic subgroup are still needed [151].

## 3. Treatments used in COVID-19 and DM: considerations

Several studies have documented and compared the effect of pharmacological therapies used in both COVID-19 patients and those for DM to reduce side effects. The general chronic inflammatory condition, the hypercoagulable prothrombotic state, and comorbidities in patients with DM require special attention when different drugs are administered. In COVID-19, corticosteroids have been used to suppress high levels of cytokines; however, they tend to cause hyperglycemia [152]. Similar situations can occur with other drugs, which can damage the heart, kidney, or liver. According to Ceriello et al. [153], medications, such as corticosteroids, hydroxychloroquine, remdesivir, and ivermectin, should be avoided or administered carefully during COVID-19 infection in diabetic patients. The administration of corticosteroids in COVID-19 patients with DM can worsen the prognosis because it influences glucose levels. Hydroxychloroquine can produce serious heart problems, which can increase the risk of complications in diabetic patients with COVID-19. The antiviral remdesivir promotes liver and kidney damage that could exacerbate the natural disorders that accompany DM, while ivermectin has not proven its efficacy against any form of COVID-19. Other studies have also noted the contrary effect of corticosteroids in treating COVID-19 in diabetic patients [154]. Hydroxychloroquine also triggers severe episodes of hypoglycemia [155]. In contrast, anti-cytokine biologicals (tocilizumab and anakinra), convalescent plasma, and monoclonal antibody therapies are better and safer without contraindications to be used in diabetic patients with COVID-19 [153].

The safety of glucose-lowering treatments has also been questioned when used in diabetic patients infected with COVID-19. An observational study in England described the associations between the prescription of different types of glucose-lowering drugs and the risk of COVID-19-related mortality in type 2 DM patients. Metformin was the main prescribed drug for DM, followed by sulfonylureas, DPP-4 inhibitors, insulin, SGLT2 inhibitors, GLP-1 receptor agonists, thiazolidinediones, meglitinides, and α-glucosidase inhibitors. Slight differences relate insulin and DPP4 inhibitors with a higher mortality risk than those who did not receive these drugs. In contrast, metformin, SGLT2 inhibitors, and sulfonylureas had a lower mortality risk than those who did not receive these drugs. The results were related to the later use of insulin during DM when patients present a more deteriorated health condition and are older. In contrast, metformin is used at the beginning of DM when patients are younger and offers fewer associated complications [156]. Lim et al. [55] presented an interesting table with recommendations of the most commonly used glucose-lowering therapies and classified them based on the scale of clinical progression severity of COVID-19 proposed by the WHO. They recommended the use of insulin for severe COVID-19 in DM patients, while metformin was recommended for diabetic patients with mild COVID-19.

## 4. Clinical differences between type 1 DM and type 2 DM patients affected by COVID-19

The severity of COVID-19 in patients with DM is tripled compared to individuals without the disease. However, reports about how COVID-19 impacts patients with type 1 DM and type 2 DM are scarce. Most of the clinical findings of COVID-19 are reported for patients with type 2 DM, likely because it has a higher frequency rate in the population. A whole-population study of England reported that only 0.4% of individuals presented type 1 DM, while 4.7% had type 2 DM. They also reported that one-third of in-hospital COVID-19-related deaths occurred in individuals with DM, of which 31.4% were in individuals with type 2 DM and only 1.5% were in those with type 1 DM [36]. Hospitalization in patients with type 2 DM has also occurred more often than in individuals with type 1 DM [157]. In addition to the frequency difference, some other factors can modify the COVID-19 impact among these two groups. Most of the cohorts of type 1 DM are 1 or 2 decades younger than those in the cohorts of type 2 DM [36],[157]–[159].

In addition, older individuals with type 2 DM usually have more associated medical issues, such as hypertension and renal complications [159],[160]. However, the outcome data adjusted for age, HbA1c, and BMI in the study of Gregory et al. [157] showed similar adjusted odds ratios for hospitalization and greater illness severity in both types of DM. Disparities by ethnicity also seem to have an effect. Ebekozien et al. [41] conducted a study with type 1 DM patients affected by COVID-19 with 44% non-Hispanic White, 30.5% non-Hispanic Black, and 25.5% Hispanics. Among them, non-Hispanic Blacks and Hispanics presented a higher median HbA1c than whites. A similar cohort formed by a total of 71% Hispanics and non-Hispanic Blacks with newly diagnosed type 1 DM and a positive test for COVID-19 was presented by Beliard et al. [161]. Vamvini et al. [162] reported that a cohort of individuals with type 1 DM affected by COVID-19 had a high percentage of non-Hispanic Black individuals (57%). In another study, Gregory et al. [157] presented type 1 DM and type 2 DM cohorts mainly formed by white people, 79% and 53%, respectively.

Diabetic ketoacidosis (DKA) is the most common reason for the hospitalization of COVID-19 patients with type 1 DM [163]. DKA appeared in some individuals with type 1 DM infected by SARS-CoV-2 [164], regularly in those with a recent diagnosis of DM [161],[165],[166], which is usual in new type 1 DM cases [167]–[169]. In patients with type 1 DM and COVID-19, non-Hispanic Blacks and Hispanics were more likely to present DKA than non-Hispanic Whites. However, after adjusting for potential confounders, non-Hispanic Black patients continued to have a greater risk [41]. Incidences of DKA have also been described in type 2 DM patients affected by COVID-19. In general, certain factors, such as male sex, older age, renal impairment, nonwhite ethnicity, high HbA1c, and previous heart failure, are associated with increased COVID-19-related mortality in both types of DM [157],[160]. However, patients with type 2 DM “are at increased risk for severe COVID-19”, while those with type 1 DM “might be at increased risk for severe COVID-19” [170].

## 5. Diabetogenic effect of SARS-CoV-2 infection

Based on information from new-onset DM in COVID-19 patients, SARS-CoV-2 infection may trigger type 1 DM by directly damaging pancreatic islet β cells. SARS-CoV-2 can trigger severe diabetic ketoacidosis at presentation in patients with new-onset diabetes [171]. Kuchay et al. [172] described three acute onset DM cases and DKA precipitated by COVID-19 in individuals with no DM history. The patients presented elevated levels of HbA1c, and their BMI indicated they were overweight. The clinical characteristics of the patients resembled type 1 DM and required multiple subcutaneous insulin injections, although after 4-6 weeks, they switched to oral antihyperglycemic drugs. Marchand et al. [173] reported a case of a 29-year-old woman who presented acute polyuria and polydipsia syndrome one month later with COVID-19 symptoms. The woman had an HbA1c level of 11.8% and was diagnosed with type 1 DM because her autoantibody against pancreatic β cells test was positive. Notably, in this case, bypass surgery was performed a year before SARS-CoV-2 infection. Suwanwongse et al. [165] presented three newly diagnosed cases of DM associated with COVID-19. A newly diagnosed case of DM was concurrent with COVID-19, and the other two newly diagnosed cases of DM occurred after the patients had COVID-19. Two had a family history of DM, and two were considered obese (BMI above 30). These authors thought that elevated HbA1c and the presence of DM risk factors, such as family history and obesity, might indicate that COVID-19 likely unmasked existing DM by aggravating their metabolic complications rather than causing DM.

Information regarding pancreatic damage induced by SARS-CoV-2 during or postinfection has emerged gradually. Autoimmune insulitis and pancreatic β-cell destruction can be triggered by viral infections, with possible type 1 DM development [174]. Specifically, the last coronavirus, SARS-CoV-1, produced acute DM by destroying pancreatic islets after ACE2 binding in the pancreas. Coincidently, SARS-CoV-2 uses a similar ACE2-mediated cell entry, leading to comparable pancreatic β-cell loss resulting from virus amplification. Clinical parameters, such as hyperlipidemia in 11.7% of COVID-19 patients, were linked to pancreatic injury caused by SARS-CoV-2 [175]. Ultrasonography of a 29-year-old man infected with SARS-CoV-2 revealed a bulky pancreas with irregular edematous margins and some peripancreatic fluid. The elevated levels of amylase and lipase, 20 and 30 times more than expected, respectively, led to COVID-19-induced pancreatitis diagnosis [176]. Similarly, the increase in amylase and lipase in six COVID-19 patients was associated with pancreatic abnormalities. The authors theorized that pancreatic injury may be related to certain factors, such as the direct effect of SARS-CoV-2 and its indirect events, including the inflammatory response, dehydration, and multiple organ dysfunction [177].

Acute pancreatitis and pancreatic necrosis have also been confirmed in patients with type 2 DM affected by COVID-19 through increased lipase serum and bulky pancreatic areas [178],[179]. Similarly, pancreatitis injury has been reported in COVID-19 patients and has been associated with additional comorbidities to DM, such as hypertension and heart disease [180]. The evidence critically reviewed by Pribadi et al. [181] showed that the increase in amylase and lipase levels in COVID-19 patients is not necessarily related to pancreatic injury due to the same increased enzymes that might be found in other clinical conditions. The exact mechanism for pancreatic injury in COVID-19 patients is unclear, and the question of whether SARS-CoV-2 can cause DM through pancreatic damage is still unclear.

Several works have revealed that the SAR-CoV-2 receptor, ACE2 protein, and other canonical entry factors, such as transmembrane serine protease 2 (TMPRSS2), are expressed in the pancreas. However, according to Coate et al. [182], the likelihood that SARS-CoV-2 directly infects pancreatic β-cells in vivo through ACE2 is reduced because they did not detect ACE2 protein in β-cells of individuals with and without diabetes. ACE2 was only seen in the exocrine tissue microvasculature and ducts of the islet. In comparison, TMPRSS2 expression was restricted to ductal cells of islets. Similar results were shown by Kusmartseva et al. [183], suggesting that SARS-CoV-2 infection of pancreatic endocrine cells, mediated by ACE2, is an improbable pathogenic event of COVID-19-related DM development. In contrast, a new approach identifies the human pancreas as a target of SARS-CoV-2 infection, suggesting that β-cell injury could contribute to the metabolic dysregulation detected in COVID-19 patients. This approach showed the expression of ACE2 and TMPRSS2 in human β-cells and the infection and replication of SARS-CoV-2 in cultured human islets. Importantly, they demonstrated that SARS-CoV-2 β-cell infection could reduce the numbers of insulin-secretory granules and impair glucose-stimulated insulin secretion. Additionally, they detected SARS-CoV-2 nucleocapsid protein in pancreatic cells through postmortem examinations of COVID-19 patients [184].

## 6. Conclusions

The severity of COVID-19 in patients with DM implies a complex interaction between these two diseases. DM presents several accompanying problems that deteriorate human health status. Severe COVID-19 outcomes (admission to the ICU/in-hospital death) in diabetic patients seem to be mainly driven by the associated complications present in the individuals at the moment of SARS-CoV-2 infection. Chronic low-grade inflammation and impaired immunity in diabetic patients aggravate the results of COVID-19 and make it more difficult for the virus to be eliminated. The nutritional status of people with DM plays a vital role in regulating additional factors that impact health conditions. A more accurate understanding of ACE2 expression and polymorphic variations in DM represents an important key to elucidating its real implication in COVID-19. The ACE2 protein is likely a target molecule for drug development or to avoid severe coronavirus disease results. The clinical findings of COVID-19 in patients with type 1 and type 2 DM are similar. The high number of fatal cases of COVID-19 in type 2 DM seems to be associated with its high prevalence, older age, and an increased proportion of comorbidities. The recently suggested potential pancreatic injury and diabetogenic effect of COVID-19 should be further studied in patients infected with SARS-CoV-2. A long-term follow-up should be performed on those patients recovering from COVID-19 to learn if its postinfection symptoms can be conducive to the development of DM.
